# A Systematic Online Living Evidence Summary of experimental Alzheimer’s disease research

**DOI:** 10.1016/j.jneumeth.2024.110209

**Published:** 2024-07-02

**Authors:** Kaitlyn Hair, Emma Wilson, Olena Maksym, Malcolm R. Macleod, Emily S. Sena

**Affiliations:** Centre for Clinical Brain Sciences, The University of Edinburgh, Edinburgh, UK

**Keywords:** Biocuration, Systematic review, Evidence synthesis, Alzheimer’s disease, Laboratory models, Reproducibility, Transparency

## Abstract

**Background:**

Despite extensive investment, the development of effective treatments for Alzheimer’s disease (AD) has been largely unsuccessful. To improve translation, it is crucial to ensure the quality and reproducibility of foundational evidence generated from laboratory models. Systematic reviews play a key role in providing an unbiased overview of the evidence, assessing rigour and reporting, and identifying factors that influence reproducibility. However, the sheer pace of evidence generation is prohibitive to evidence synthesis and assessment.

**New method:**

To address these challenges, we have developed AD-SOLES, an integrated workflow of automated tools that collect, curate, and visualise the totality of evidence from in vivo experiments.

**Results:**

AD-SOLES is a publicly accessible interactive dashboard aiming to surface and expose data from in vivo experiments. It summarises the latest evidence, tracks reporting quality and transparency, and allows research users to easily locate evidence relevant to their specific research question.

**Comparison with existing methods:**

Using automated screening methodologies within AD-SOLES, systematic reviews can begin at an accelerated starting point compared to traditional approaches. Furthermore, through text-mining approaches within the full-text of publications, users can identify research of interest using specific models, outcomes, or interventions without relying on details in the title and/or abstract.

**Conclusions:**

By automating the collection, curation, and visualisation of evidence from in vivo experiments, AD-SOLES addresses the challenges posed by the rapid pace of evidence generation. AD-SOLES aims to offer guidance for research improvement, reduce research waste, highlight knowledge gaps, and support informed decision making for researchers, funders, patients, and the public.

## 1 Background

Alzheimer’s disease (AD) is a devastating neurodegenerative disorder characterised by progressive cognitive decline and memory loss. By 2050, it is estimated over 100 million people will be living with the condition worldwide (Brookmeyer et al., 2007). As the global population ages, AD continues to weigh heavily on healthcare systems, society, and the wider economy. Over the last two decades, many billions have been spent on laboratory research conducted across the pharmaceutical industry and academic institutions in concerted efforts to develop disease-modifying treatments (Kim et al., 2022). The use of preclinical models has helped deepen our understanding of disease aetiology and has enabled researchers to evaluate thousands of potential therapeutic compounds for safety and efficacy prior to testing in humans. Positive data from preclinical trials have encouraged numerous clinical trials; unfortunately, nearly all therapies tested have failed to demonstrate significant therapeutic benefit for those living with AD (Pistollato et al., 2020; Zahs and Ashe, 2010; Cummings et al., 2014). To illustrate the scale of the problem, it has been estimated that since the millennium, over 400 trials testing AD targeted therapeutics have failed (Rinaldi, 2018). The recent Food and Drug Administration approval of two monoclonal antibody therapies, Lecanemab and Aducanumab is an encouraging development. However, their “real world” efficacy is still to be evaluated, and both approvals have been accompanied with controversy over the strength of the evidence justifying their use (Mullard, 2021; Reardon, 2023).

When planning laboratory experiments to investigate AD pathology or evaluate new therapeutic target, it is important to consider how the resulting data will fit into the broader context of existing knowledge. Thought leaders in AD emphasise the need for robust and reproducible target validation to facilitate drug discovery (Mauricio et al., 2019). Rather than relying on evidence from a single study, we should seek incremental evidence from a range of experiments that attempt to answer the same and related research questions across different laboratories and model systems. AD is a complex, multifactorial disorder. No model can be fully representative of the human condition, though some may be more relevant for investigating specific aspects of the disease (e. g. tau pathology) than others (Shineman et al., 2011), and could provide mechanistic insights into how these facets of the disease manifest (McGonigle and Ruggeri, 2014). As pointed out by others (Quinn, 2018), 15 new therapies have been identified over the last decade to treat multiple sclerosis (MS) – another highly complex neurological disease – which indicates that lack of a “perfect” animal model doesn’t prevent progress. In MS, there have been efforts to selectively target the inflammatory aspects of the disease which are reproduced in animal models (Constantinescu et al., 2011), and successful clinical trials have appropriately aligned their outcomes with preclinical efficacy studies.

By improving our understanding of the quantity and quality of existing research, we could significantly reduce research waste. By taking stock of the literature in its entirety, we could prevent the un-necessary duplication of experiments. Furthermore, by combining effect sizes across different studies measuring similar outcomes, we can develop recommendations on the sample sizes required in different contexts for adequate statistical power (Currie et al., 2018). Through examining gaps in our existing knowledge, we can ensure that we prioritise and fund experiments which are likely to be of most scientific value. However, in practice, the approach to identifying prior studies is often haphazard and too reliant on the journals that we subscribe to, the conferences we attend, or the study findings shared within our networks.

Systematic reviews and meta-analyses seek to provide an unbiased overview of the evidence, assess rigour and reporting, and identify which experimental design factors may influence reproducibility and predictive value (Hooijmans and Ritskes-Hoitinga, 2013; Sena et al., 2014). Taking a systematic approach can facilitate a deeper understanding of what makes research reliable, how it can most effectively be improved, and promote more informed decision-making (Macleod et al., 2014; O’Hagan et al., 2018). Across biomedical research, most pre-clinical systematic reviews have focused on the internal validity of in vivo experiments modelling human diseases. Past work has identified methodological weaknesses and poor reporting quality (where studies do not report the details of experimental design, conduct, and analysis). Persistent failures to report such measures have been associated with inflated estimates of treatment efficacy and likely lead to false positive results, where a drug appears to improve outcome but in reality does not (Bello et al., 2014; Hirst et al., 2014; Tsilidis et al., 2013). Previous work has highlighted the extent of the problem within the in vivo preclinical AD literature (Veening-Griffioen et al., 2019; Egan et al., 2016; Snyder et al., 2016; Sukoff Rizzo et al., 2020; Chakroborty et al., 2022), with poor reporting of key experimental design characteristics and measures to reduce the risk of bias. In a retrospective review of the preclinical evidence that informed six high-profile AD clinical trials (Karran and Hardy, 2014), the authors concluded that some were “very unlikely to succeed” based on the prior evidence. Of those reviewed, 4 (Tramiprisate, Semagacestat, Bapineuzumab, Solanezumab) had incomplete or inconsistent in vivo data from animal models and 2 had in vivo data which did not support progression to phase 1 clinical trials (Tarenflurbil, Gammagard). Although some of the pitfalls of the compounds were known at the time, a thorough and rigorously conducted systematic review of the evidence may have provided clear guidance about where the gaps were, how strong the evidence was for a specific outcome to be measured in patients (e.g. the ability to reduce levels of existing amyloid plaques or cognitive improvements), and the likelihood of clinical benefit.

In recent years, we have endeavoured to perform wide-ranging and comprehensive systematic reviews of the in vivo and more recently in vitro literature, often retrieving tens of thousands of potentially relevant citations from biomedical database searches (Bannach-Brown et al., 2021). We have observed that the specific animal or cell-based model(s) used and outcome(s) evaluated are not always clear without reading the full text of a published article, due to insufficient detail in the title, abstract, and other searchable fields (Hair, 2022; Wilson et al., 2023a). This can result in a trade-off between retrieving too many irrelevant studies and missing potentially important studies. In highly research-intensive fields, including AD, the pace of evidence generation plus the time, expertise, and resources required to complete such a review presents likely presents a significant barrier to systematic scientific advancement. After billions of pounds, millions of animals, and thousands of experiments, there is a huge body of potentially useful data dispersed across the literature. Curating these data in a form which allowed them to be accessed and exploited quickly, with minimal manual effort, could be transformative.

Harnessing technological advancements such as natural language processing and machine learning (Bannach-Brown et al., 2021), we have developed an integrated workflow of automated tools to systematically collect, curate, and visualise evidence from in vivo experiments. Systematic Online Living Evidence Summaries, or SOLES projects (Hair et al., 2023), are available as publicly accessible interactive dashboards, refreshed with new evidence on a regular basis. Here, we describe the development of AD-SOLES; a dashboard to accelerate evidence-driven preclinical research in AD models. Using the dashboard, all AD research stakeholders including researchers, funders, patients, and the public can gain a better understanding of the quantity and quality of the existing evidence. We intend that AD-SOLES be a platform to support (i) research-on-research (including systematic reviews) of in vivo AD models, (ii) research improvement activities, and (iii) evidence-based decision making.

## 2 Methodology

### 2.1 Automated citation retrieval

We retrieve relevant citations from across three biomedical sources: PubMed, Web of Science (WoS), and Scopus. Instead of limiting our search to include specific models or in vivo research, we use broad and simple search terms (Table 1) to identify all potentially relevant research related to Alzheimer’s disease. This is partly due to an uncertainty in whether citations have been indexed in enough detail to selectively retrieve experiments in animal models, and to preserve AD studies in in vitro and clinical populations for future expansions of AD-SOLES. On a weekly basis, new citations are retrieved programmatically using modified versions of existing R packages to query WoS (*wosr* (Barnier, 2020)), *Scopus* (*ScopusAPI* (Belter, 2021)), *and Pubmed* (*RISmed* (Kovalchik, 2021)). Each tool uses application programming interfaces (APIs) to find and retrieve relevant citations. We modified each function to format the retrieved data and retain the most important metadata (including title, authors, abstract, DOI, pages, volume, issue, journal, URL, and database accession numbers).

Once new citations are identified, we use the Automated Systematic Search Deduplicator (ASySD) (Hair et al., 2023) to remove any duplicate copies of citations. Citations from the previous two months of search results are also compared by ASySD to remove any citations that have been retrieved previously. Once complete, the unique set of new citations are added to the AD-SOLES database. To capture any additional duplicate citations that have been missed, we also perform a comprehensive deduplication process (using automated and manual deduplication functions in ASySD) every 6 months.

### 2.2 Screening for in vivo research

Using screening decisions from human reviewers, we trained a machine learning algorithm hosted at the EPPI Centre, University College London, having applied this tool successfully in previous systematic reviews (Currie et al., 2018; Bannach-Brown et al., 2019) and classification tasks (Wilson et al., 2023a). To train the algorithm for our classification task, we collated 4182 verified screening decisions (where at least two human reviewers were in agreement about whether a publication did or did not include reports using an in vivo AD model from four systematic review projects: an earlier attempt to create a living systematic review of AD models (Hair, 2018), an ongoing review of Open Field Test measurements in animal models of AD (Hair and Sena, 2021), an ongoing review of in vitro slice electrophysiology measurements in AD models (Hair et al., 2021b), and an older review of interventions in transgenic AD models (Egan et al., 2016). Human decisions were sent to the machine learning algorithm alongside their corresponding titles and abstracts. Each time new publications are retrieved, the machine algorithm is re-trained and applied, leading to marginal differences in performance between each iteration. We keep a log of performance (see Table 2 for performance metrics used) with unique identifiers for each run. This is to ensure we can track any significant changes over time that may suggest we need to generate more training data to improve performance.

### 2.3 Retrieving full texts

To retrieve full texts (either in PDF, XML, or text format), we use the DOI of included studies to query the Unpaywall (Orr et al.) and CrossRef ([Available from: ⟨https://www.crossref.org/⟩]) APIs and retrieve downloadable links to open access full texts. We also make use of Elsevier (Elsiever TDM API) and Wiley (Wiley TDM API) APIs to programmatically access and download additional full texts available via our institutional subscriptions (University of Edinburgh).

### 2.4 Study feature tagging

To tag each publication by animal model(s), outcome measure(s), intervention(s), species, and sex(es), we developed customised dictionaries of regular expressions or “regex” patterns, which are highly specialised to search bodies of text for specific instances of characters, words, and phrases (Bui and Zeng-Treitler, 2014). All regex dictionaries used within AD-SOLES are available on the Open Science Framework (OSF) at https://osf.io/yhxq4/. For any future updates, we will upload a versioned file to this OSF project.

#### 2.4.1 Model dictionary

We extracted a list of models identified in a previous review of transgenic Alzheimer’s disease models (Egan et al., 2016). We supplemented this list with a curated database of transgenic models and alternative names available via the Alzforum website (Alzforum Model Database). We first converted the list of models into regex strings using an in-house R script. This conversion included added word boundaries between each word (to ensure that “APP” didn’t match with “PAPP”) and placing Boolean “OR” operators between each possible variation for each model (to ensure that 3 × Tg or 3 × TG or 3 × Tg-AD signalled a match for the 3 × Tg-AD model. Early validation results indicate that there are often matches with references to other work mentioning a specific model. In an attempt to improve this, we created a regex to extract model sentences and applied the regex dictionaries for model, sex, and species to those sentences.

#### 2.4.2 Intervention dictionary

Across SOLES projects, we use a list of over 12,000 compounds obtained from DrugBank (Wishart et al., 2006) which has been programmatically converted into regular expressions. For AD-SOLES, we also extracted a list of interventions, target types, and drug classes from the Alzforum website (Alzforum Therapeutic database) and developed regexes for each drug to capture synonyms, alternate spellings, and punctuation differences.

#### 2.4.3 Outcome dictionary

We extracted a list of behavioural outcomes identified in a previous review of transgenic Alzheimer’s disease models (Egan et al., 2016) and converted these terms to regular expressions through manual review of studies in this annotated dataset to check for variations in language. For example, the “Morris water maze” may also be called “water maze” or “MWM” or “Morris maze”. We also developed additional regular expressions to support an ongoing review of in vitro hippocampal slice electrophysiology in AD models (Hair et al., 2021b).

#### 2.4.4 Sex dictionary

We developed a simple regular expression pattern for male and female animals.

#### 2.4.5 Species dictionary

We developed simple regular expression patterns for the most commonly used animals in neurodegeneration research.

#### 2.4.6 Model sentence extractor

A specialised regex pattern was also created to extract sentences within a publication containing a description of the animal model describing where the model was obtained from and/or details of model generation. Regex dictionaries for model sex, and species were then applied to this extracted text directly, with the aim of improving specificity compared to full text performance.

### 2.5 Study feature tagging: validation

To estimate the usefulness of feature tagging and determine the optimal approach, we performed a validation study. We applied regex dictionaries to the title/abstract/keyword fields (tiabkw method) and full text of each included study in AD-SOLES. For model, sex, and species tagging, we also extracted model sentences and applied regex dictionaries to the extracted text. When applying regex dictionaries for interventions throughout the development of SOLES projects, we have become aware that there are often many spurious matches within the full text due to non-specific drug synonyms and uses in other contexts. For example, a compound could be used as an intervention in one study and as a culture medium in another. At present, the intervention dictionary is not specific enough for use on full texts. For this reason, we only apply intervention regex dictionaries using the tiabkw method.

We collated citations in AD-SOLES which had been tagged with at least one model, outcome, sex, and species using multiple approaches. From the fully tagged studies, we obtained a random subset of 100 articles to manually check. A single reviewer read the full text of each study and checked whether each of the identified tags were accurate or not, providing a TRUE/FALSE decision beside each tag on a google sheets spreadsheet. Following this, decisions were imported into R for analysis. It is not possible to calculate the true sensitivity of a regex approach using the approach described here. For example, it is unclear how many studies using a certain model in the AD-SOLES database have not been tagged. Instead, we estimated the positive predictive value (precision), and specificity of each approach for each tag type. We also estimated the sensitivity for different approaches based on the validated model, sex, outcome, species, and intervention tags that had been identified in the subset. In other words, for all of the validated model tags across the 100 studies, what proportion were correctly identified using only the tiabkw approach?

To identify optimal approaches going forward, we also aimed to compare full text regex match frequencies at different thresholds (1 match or more, > 1 matches, > 2 matches), 1 or more title/abstract/keyword matches, and 1 or more model sentence matches. Optimal approaches were defined as having a precision of > 0.80, indicating that when a study was tagged, there was an 80 % likelihood that the tag has been correctly applied. Where there are multiple approaches with similar precision, we will preferentially select for the one with a higher sensitivity.

### 2.6 Transparency assessments

To obtain estimates of data sharing and code sharing practices across the AD literature, we employed the ODDPub tool (RRID:SCR_018385) developed to support automated open data detection in biomedical research articles (Riedel et al., 2020). ODDPub was previously validated on randomly sampled publications from PubMed and had an estimated sensitivity of 0.73 and a specificity of 1.0. Any articles in PDF format were converted before running the tool, as ODDPub requires articles in text format for processing. To obtain the open access status of publications, we queried the CrossRef database using rcrossref R package (Chamberlain et al., 2020) using the DOI of included articles.

### 2.7 Risk of bias assessment

To assess risk of bias reporting, we developed an automated tool for use in preclinical experiments (Wang et al., 2022). The tool uses natural language processing models to provide a probability score on the following measures to reduce the risk of bias: (1) random allocation to groups, (2) blinded outcome assessment, (3) conflict of interest statement, (4) compliance with animal welfare regulations, and (5) reporting of animals excluded from the analysis. Probability scores of greater than 0.5 indicate that a measure is reported. The tool is python based (Wang, 2021), and we implemented this into our R based workflow using the reticulate R package. This tool was previously validated and found to achieve F1 scores of 0.82, 0.82, 0.83, 0.91, and 0.47 for random allocation, blinded outcome assessment, conflict of interest statement, compliance with animal welfare, and reporting of exclusions respectively.

### 2.8 Additional metadata

Often, newly identified citations may lack abstracts, DOIs, or other important metadata. To retrieve information that is missing form a citation record, we pull additional metadata from CrossRef and Open-Alex (Priem et al., 2022) databases (via openalexR (Maloney, 2022) and rcrossref (Chamberlain et al., 2020)). We also use OpenAlex to maintain a record of retracted studies.

### 2.9 A “living” workflow

Each week, we run an R script containing each step of the workflow to retrieve, screen, and tag new evidence as it emerges. Newly curated datasets are sent to the underlying AD-SOLES database, which feeds into the web application in real time. In this way, we are able to continually refresh AD-SOLES with minimal human effort.

### 2.10 Web dashboard

We created a web application using R Shiny to allow users to visualise, interrogate, and download subsets of the AD-SOLES database. The code underlying the shiny application is available on GitHub (Hair, 2023) and the website is openly accessible at: https://camarades.shinyapps.io/AD-SOLES/.

### 2.11 Data integrity and version control

All data is stored in a Postgres SQL database hosted on Amazon Web Services. Starting from June 2023, we deposit weekly database snap-shots on the Open Science Framework following each search update (available at https://osf.io/8r3p7/).

## 3 Results

### 3.1 Research included in AD-SOLES

**Fig. 1:**
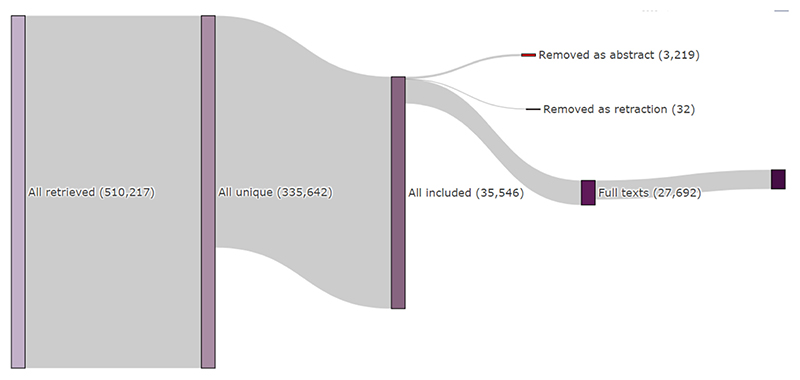
Sankey flow diagram of publications currently in AD-SOLES database.

As of this date (8th June 2023), we have retrieved a total of 510,217 citations from across biomedical databases, of which 335,642 were considered by ASySD to be unique (see Fig. 1). Following classification by the machine learning algorithm, 35,546 studies are included in the database. 3219 publications were removed from our pipeline as they are highly likely to be abstracts only, and 32 were removed as they were retracted. Of these included publications, we were able to retrieve 27,692 (77.8 %) of the full texts.

**Fig. 2:**
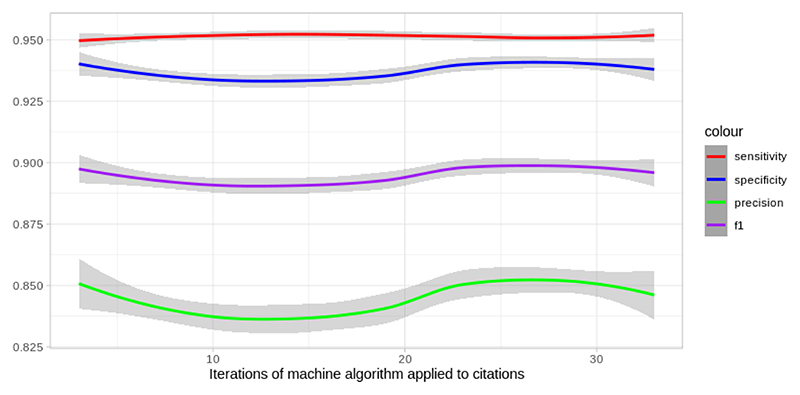
Performance metrics of machine algorithm for in vivo AD research over subsequent iterations.

The machine classifier performs with an average sensitivity of 95.1 % and an average specificity of 93.7 %. Fig. 2 shows performance across different machine classifier runs (#1 representing the 1st time the classifier was applied and #30 representing the 30th run).

**Fig. 3:**
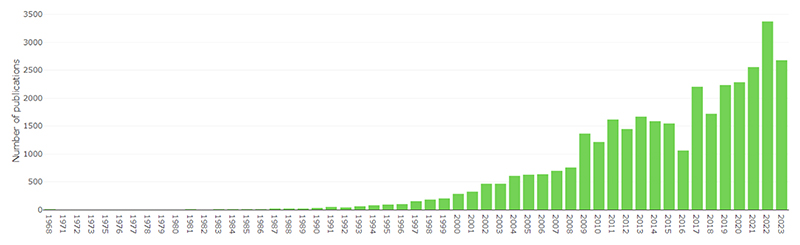
Number of citations included in AD-SOLES per year.

The pace of publication of research in in vivo Alzheimer’s disease models has grown substantially over time (Fig. 3). There were 1221 new articles in 2010, 1741 in 2015, and 2312 in 2020. Since the start of 2023, we have already retrieved 2679 new included articles (79.3 % of 2022’s total) as of 8th June 2023.

### 3.2 Study tagging validation

The random sample of 100 studies used to validate study tagging approaches were derived from a subset of 2837 included studies that had at least one tag for model, sex, species, and outcome using each method (full text, tiabkw, model sentence) and had a tag for an intervention using the tiabkw method. Of the 100 selected studies, 2 were excluded from the tagging validation as they were conference abstracts (identified by human reviewers). Within each of the 98 studies assessed, there were often several classifications applied (e.g. multiple models). In total, across all tagging methods there were n = 352 (model), n = 134 (sex), n = 190 (species), and n = 240 (outcome) tags identified. For drugs, using only title, abstract, and keyword fields, we identified n = 202 tags.

Overall, tiabkw matches were less sensitive than other methods but highly specific i.e. if a model was mentioned in the title, keywords, or abstract, it was highly likely to be used for the experiments. Full text matches and model sentence matches were less specific, but are likely to be useful in addition to tiabkw matches for enhancing sensitivity.

For model, sex, and species, the best performing approaches were the tiabkw regex and searching within the extracted model sentence (Table 3). For model, the precision of the model sentence method was not as high as expected (0.793) but deemed good enough for application in AD-SOLES. For outcome measure, more than one mention in the full text or one or more mention using the tiabkw method were the most favourable approaches. The logic underlying the AD-SOLES application was guided by the optimal approaches (Table 4).

### 3.3 Models, outcomes, and interventions

**Fig. 4:**
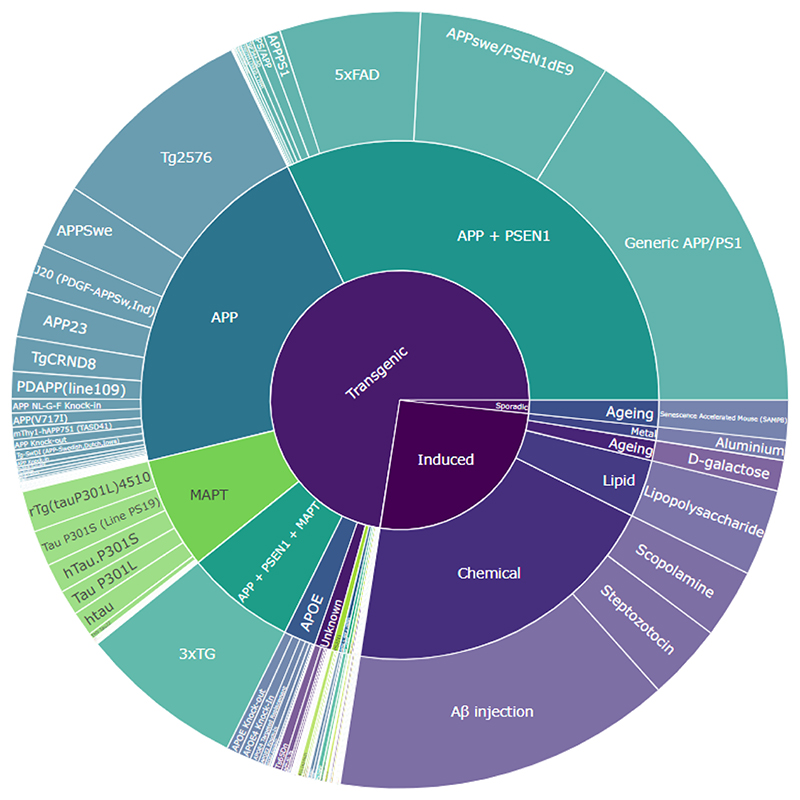
Sunburst plot of models in AD-SOLES. Segment indicates the relative proportion of the literature in that category.

**Fig. 5:**
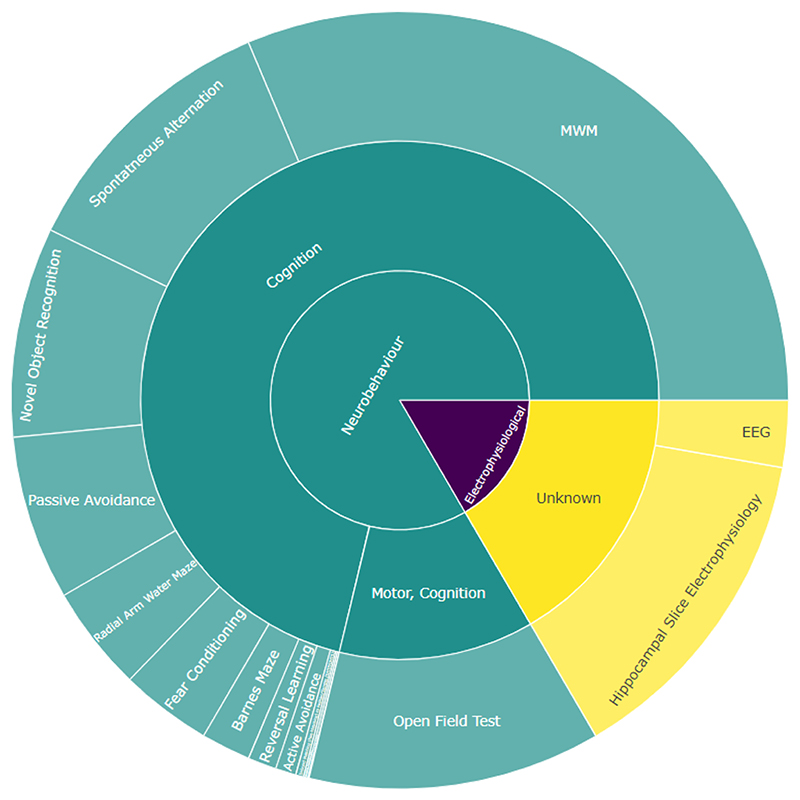
Sunburst plot of outcomes in AD-SOLES. Segment indicates the relative proportion of the literature in that category.

**Fig. 6:**
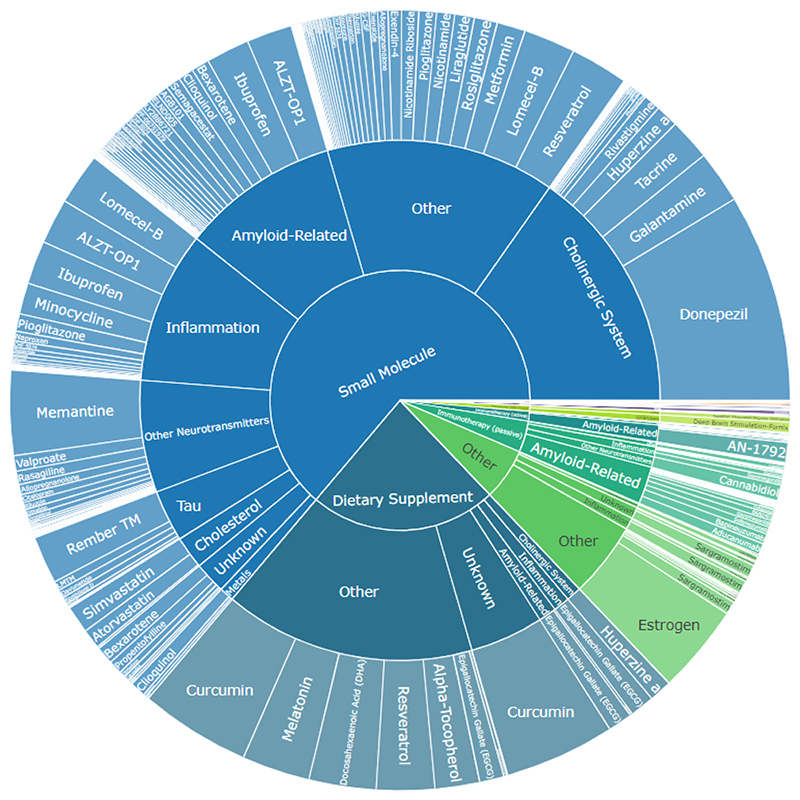
Sunburst plot of interventions in AD-SOLES. Segment indicates the relative proportion of the literature in that category.

Of the 35,546 studies included, 20,670 (58.2 %) 16,390 (46.1 %), and 20,446 (57.6 %) have been successfully tagged with at least one model, outcome, and intervention respectively using the optimal approaches. Sunburst plots visualising the number of publications in each category are shown in Figs. 4–6. The most common model is APP/PS1 (Fig. 4) described as “Generic APP/PS1” in the SOLES platform due to inability to distinguish between different APP/PSEN1 mutation models. The Morris Water Maze is the most commonly measured behavioural outcome (Fig. 5), while Donepezil is the most commonly used treatment (Fig. 6).

**Fig. 7:**
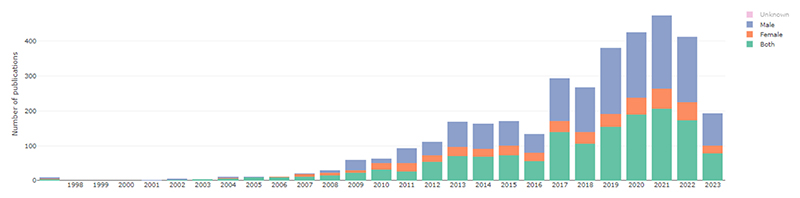
Articles with APP/PSEN1 models stratified by sex of animals in AD-SOLES. Note: The bar height reflects a downward trend in use of APP/PS1 models between 2021 and 2023, while the colour fill shows the number of publications using male and female animals.

In the most commonly used model category (transgenic mice with APP + PSEN1 mutations), we see a continued preference to use male animals or a combination of male and female animals within experiments (Fig. 7).

**Fig. 8:**
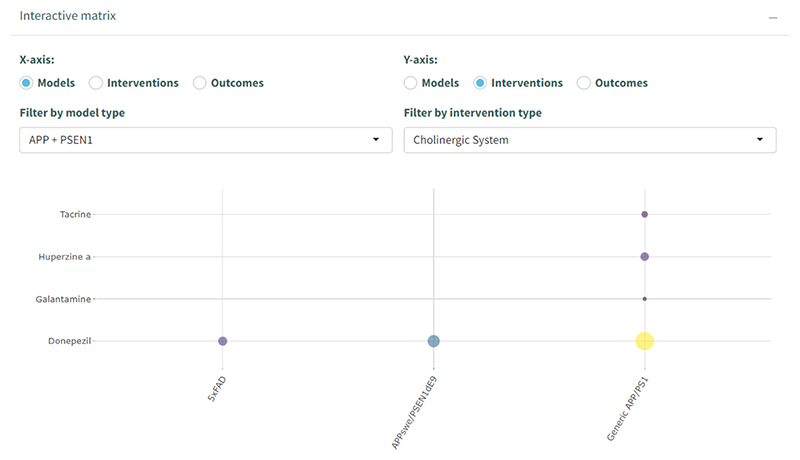
Matrix gap map with drugs targeting the Cholinergic system tested on APP/PSEN1 models in AD-SOLES.

Using the interactive matrix functionality within AD-SOLES, it is possible to visualise overlap across tags. For example, looking across interventions which target the Cholinergic system (Fig. 8), Donepezil has been tested in 5 × FAD, APPSwe/PSEN1de9, and APP/PS1 (generic) models, while Tacrine and Huperzine A, and Galantamine have only been tested in a small number of experiments in APP/PS1 models.

### 3.4 Study quality and transparency

**Fig. 9:**
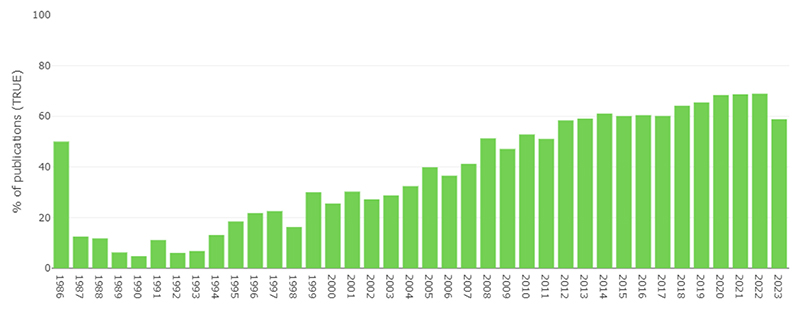
Number of open access publications over time in AD-SOLES. Green bars indicates that a paper is open access; grey indicates closed access. Data source: CrossRef linkage with n= 31,245 articles.

At present, 31,245 out of 35,546 (87.9 %) citations were findable (via DOI) in the CrossRef database. The proportion of open access publications has increased considerably since 2008 (Fig. 9). Overall, 57.5 % of publications in this dataset are open access. Stratifying open access (OA) status by type, we see the options of green OA (depositing accepted manuscript in open repository) and bronze OA (available via publisher but not formally licenced for re-use) publication became less popular, while gold OA (immediate, unrestricted access) publishing gained traction (Fig. S1).

ODDPub was applied successfully to 26,920/35,546 articles. Overall, open data statements were identified in 6.3 % of articles, while open code statements were identified in 0.6 % of articles. Since 2015, there has been a year-on-year increase in open data sharing in this literature (Fig. S2).

**Fig. 10:**
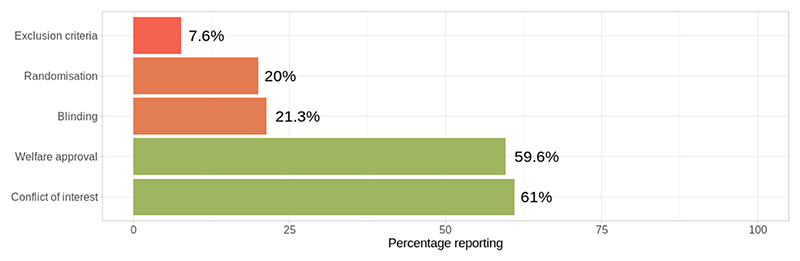
Overall percentage of publications reporting key measures to reduce the risk of bias in AD-SOLES. Tool: ROB preclinical tool (Wang et al., 2022) applied to N = 21,980 full text articles.

Currently, 21,980/35,546 articles in the AD-SOLES database have been assessed for risk of bias reporting. A subset of full texts have not yet been assessed due to large file sizes (> 50,000 bytes) causing memory issues when processing full texts. Reporting of conflict of interest statements (61.0 %) and welfare approval (59.6 %) was moderate across the dataset, while reporting of randomisation to experimental groups and blinded outcome assessment was low (20.0 % and 21.3 % respectively). Very few studies were found to have reported exclusion criteria for animals/datapoints (7.6 %). Fig. 10

### 3.5 Downloading relevant research

Using the study tags, research users can download relevant citation lists from within the web application. The searchable study table (Fig. S3) contains all of the citations present in the AD-SOLES database with associated metadata (Year, Author, and Title, with a link to the publication if available). Study feature tags which have been applied to each citation are also visible. Users are able to search the title, abstract, and keywords of included studies using Boolean AND/OR logic and filter results by model, intervention, outcome measure, and year of publication. To support the need for systematic reviews where it is essential a study is not missed, we have a “high sensitivity” toggle that can be switched on to include studies where an intervention, model, or outcome is mentioned anywhere within the full text.

## 4 Discussion

### 4.1 Data curation to support evidence-based research and discovery

Based on our analysis of the AD-SOLES dataset, the evidence from AD animal models is continually expanding, presenting a mounting challenge for research users to keep up –to date with the emerging data. To address this, we have developed AD-SOLES to harness the full potential of existing data to inform future research; an openly available dashboard with an integrated workflow of automated tools to support curation. To date, we have identified over 35,000 publications likely to contain experimental data from AD models. Through synthesising this vast evidence base, we can reduce the burden on basic scientists to continually stay up to date with the latest research developments relevant to their line of enquiry. Using AD-SOLES, we aim to make it easier to grasp the quantity and quality of existing evidence in a specific animal model; for a specific intervention; or measured on a given intervention.

We envisage benefits not only for laboratory researchers, but for research funders, charities, and other stakeholders who need to stay up –to date with the current research landscape. Those conducting literature reviews of specific areas (for example, a PhD student starting a research project, or a team of researchers aiming to conduct a meta-analysis) can use AD-SOLES as an accelerated starting point, enjoying the benefits of automation without the need to apply machine learning or other automation approaches themselves.

Going forward, we aim to expand AD-SOLES to encompass both in vitro and clinical data relating to Alzheimer’s disease pathology and potential drug targets. It has been argued that promoting reproducibility of preclinical research alone is not sufficient, and that we need to triangulate the evidence from multiple approaches to answer one question (Munafò and Davey Smith, 2018). Through integration with drug discovery databases and the application of our existing natural language processing tools, we will look to map pathological concepts and drug targets across different levels of evidence to support target validation. Given the sheer volume of data available, we will leverage technological advancements to identify patterns and insights that would be challenging to identify manually.

### 4.2 Continuous monitoring of rigour and transparency

Through continuous monitoring of key aspects of rigour and reporting quality over time, AD-SOLES could provide insights into areas where improvement is most needed. From the data currently available, it is clear that measures to reduce the risk of bias are severely under-reported, in agreement with other recent reports in the preclinical AD literature (Chakroborty et al., 2022). To facilitate efficient and cost-effective drug development, it is important that experiments are adequately powered and rigorously conducted to reach the correct conclusions on efficacy (Gulinello et al., 2018). It is important to note that a failure to report does not necessarily determine that an experiment was conducted without appropriate controls. However, by taking steps to improve conduct and reporting, we can more adequately assess the strength of the cause and effect relationship (internal validity) within an experiment.

Issues around the animal model used and the context they are being used in being too different from the human condition may also contribute to translational failures (Perry and Lawrence, 2017). In the dashboard currently, it clear that male rodents continue to be employed preferentially over female rodents for modelling AD. Variations in husbandry conditions, background strains, genetics, age, comorbidities, and a host of other factors may impact upon results (Wilson et al., 2023b; Justice and Dhillon, 2016). In future, we anticipate integrating tools to extract other important methodological details pertaining to the rigour, and transparency of AD experiments into the AD-SOLES pipeline. Efforts will concentrate around key criteria, such as those laid out in specialised guidelines developed to improve preclinical design and efficacy in AD (Snyder et al., 2016) and the latest version of the Animal Research: Reporting of In Vivo Experiments (ARRIVE) guidelines. This could, in future, feed into to the development of specialised “living” guidelines (Akl et al., 2017), research improvement targets, and initiatives to maximise the validity, transparency, and reproducibility of in vivo experiments, while minimising research waste.

The dashboard currently shows an upwards trajectory in open-access publications, with a greater proportion being fully open access than ever before. However, it seems that data sharing and code sharing are still not commonplace. In light of the recent AB*56 controversy, where a prominent researcher was accused of fabricating data derived from AD mouse models (Piller, 2022), transparency in how an experiment was conducted and analysed is paramount. Over the entire dataset, it is concerning that less than 1 % of publications reporting open data or open code availability statements. However, it is extremely encouraging to see the continual trend of increasing data sharing in over the last 10 years (from 3 % in 2013 to 18 % of the dataset in 2023).

Through integration with OpenAlex, we hope to expand the capabilities of AD-SOLES to monitor the impact of research funded from different sources. As others have suggested (Pistollato et al., 2020), the vast amounts of grant funding provided for hundreds of translational AD projects in experimental models could be retrospectively evaluated to inform future decision making and encourage a more open dialogue between research stakeholders on the uptake of open research practices.

### 4.3 Reciprocity with systematic reviews and curation efforts

The development of AD-SOLES has relied heavily on existing annotated datasets from preclinical systematic reviews of Alzheimer’s animal models. If automated tools are to perform optimally, on a par with a human reviewer, tool developers need as much training data as possible. Going forward, we hope to initiate a reciprocal relationship with researchers who conduct systematic reviews using our curated datasets. Annotated data could be fed back into the database, to be used for future tool development and validation. Where possible, we will also seek to align, integrate, and collaborate with other initiatives to curate dementia research, such as AlzPed (Chakroborty et al., 2022), a manually curated database of over 1000 preclinical AD experiments.

Given the utility of systematic reviews to identify new research avenues and guide research progress, we hope to foster and encourage a greater uptake of these approaches in the preclinical AD literature.

### 4.4 Limitations

An important consideration in the AD-SOLES workflow is the omission of potentially relevant records at multiple points. With the current machine classifier performance, we expect to include ~95 % or relevant research and exclude > 90 % of irrelevant research. This trade-off was deemed necessary to ensure that the metrics displayed on the dashboard were specific to the AD literature. However, this could pose an issue under some circumstances when relevant records have not entered into the pipeline. In the near future, we plan to manually screen subsets of the publications which fall close to the boundary of inclusion to provide additional edge-case data to train the classifier. Currently, over 30 % of relevant publications do not have openly accessibly full-texts, or full-texts which are not accessible under our institutional subscriptions. This prevents us from applying automated natural language processing tools to determine risk of bias reporting, open data reporting, and other study characteristics. There is still some way to go before all full-texts will be fully accessible via automation technologies.

The tagging of studies by model, intervention, and outcome measure requires additional validation and improvement to reach optimal performance. Most studies are not fully tagged, which may reflect that those experiments have experimental features which are not on our list, that the regex approach isn’t sensitive enough to detect all instances, or that those studies are less relevant or do not use AD models. Many elements, including pathological outcome measures, non-rodent models, and novel therapeutics are not sufficiently managed by our current approach. While employing regular expression dictionaries is suitable for cases with limited variations, leveraging natural language models (NMLs) such as PubMedBERT (Gu et al., 2021) holds promise. Recent work to identify chemical entities within AD research found that significant performance gains were made when NLMs were combined with a dictionary-based approach (Mullin et al., 2023). We recently developed and validated a NLM-based preclinical PICO (population, comparator, intervention, outcome) tool for this purpose (Wang et al., 2021). We plan to validate both dictionary and natural language processing-based methods for the extraction of key study characteristics versus gold standard human annotations. In the future, the widespread adoption of recognised ontology terms and identifiers such as research resource IDs (Bandrowski and Martone, 2016), mouse genome database identifiers, or strain numbers would simplify this process and reduce reliance on increasingly sophisticated language models. Alternatively, requesting researchers to tag studies with a predefined list of characteristics during journal submission or publication could achieve a similar outcome.

Comparable to other software tools, the AD-SOLES pipeline and dashboard will require ongoing maintenance to ensure it remains usable, accessible, and up to date. We will engage with dementia research users and stakeholders to determine where more development is required, and will seek to acquire long-term funding to support sustainability.

## 5 Conclusion

AD-SOLES aims to provide a valuable resource for researchers, funders, and other stakeholders in the AD research community. Through the use of AD-SOLES, we hope to facilitate evidence-based research, promote rigour and transparency, and foster collaborative evidence synthesis projects within AD research.

## Supplementary Material

Supplementary material

## Figures and Tables

**Table 1:** Simplified AD search terms for retrieving research for AD-SOLES.

Database/source	Search terms
Web of Science	TS = "Alzheimer”
Scopus	“Alzheimer*”
PubMed	Alzheimer Disease [All Fields] OR "alzheimers disease"[All Fields] OR alzheimer*[All Fields]

**Table 2:** Performance metrics for machine classifier.

Metric	Description
Sensitivity	Proportion of correctly included citations: $\frac{\;true\;positive\;}{\;true\;positive\;\text{+}\;false\;negative\;}$
Specificity	Proportion of correctly excluded citations: $\frac{\;true\;negative\;}{\;true\;negative\;\text{+}\;false\;positive\;}$
Precision	Proportion of machine predictions correct: $\frac{\;true\;positive\;}{\;true\;positive\;\text{+}\;false\;positive\;}$
F1	Harmonic mean of precision and recall: $2•\frac{\;precision\;•\;sensitivity\;}{\;piectston+sensututy\;}$

**Table 3:** Results from AD-SOLES study tagging validation.

Type	Method	Specificity	Sensitivity (versus all methods)	Precision	TP	FP	TN	FN
**Model**	full text > 0	0.005	1	0.404	143	208	1	0
	full text > 1	0.45	0.909	0.526	130	115	94	13
	full text > 2	0.746	0.692	0.652	99	53	156	44
	tiabkw	0.933	0.727	0.883	104	14	195	39
	model sentence	0.861	0.762	0.793	109	29	180	34
**Sex**	full text > 0	0.053	1	0.865	115	18	1	0
	full text > 1	0.579	0.678	0.907	78	8	11	37
	full text > 2	0.789	0.417	0.923	48	4	15	67
	tiabkw	0.895	0.522	0.968	60	2	17	55
	model sentence	0.632	0.991	0.942	114	7	12	1
**Species**	full text > 0	N/A	1	0.521	99	91	0	0
	full text > 1	0.143	1	0.559	99	78	13	0
	full text > 2	0.407	0.626	0.534	62	54	37	37
	tiabkw	0.967	0.98	0.97	97	3	88	2
	model sentence	0.802	0.98	0.843	97	18	73	2
**Outcome**	full text > 0	N/A	1	0.671	161	79	0	0
	full text > 1	0.646	0.932	0.843	150	28	51	11
	full text > 2	0.848	0.497	0.87	80	12	67	81
	tiabkw	0.987	0.422	0.986	68	1	78	93
**Intervention**	tiabkw	N/A	N/A	0.417	85	117	N/A	N/A

TP: true positive, FP: false positive, TN: true negative, FN: false negative. N/A cells indicate that the measure cannot be calculated. Rows highlighted in blue indicate optimal approaches.

**Table 4:** Optimal logic for AD-SOLES study tagging.

Tag element	Frequency: title, abstract, keywords	Frequency: full-text
**Model**	≥ 1	≥ 1 in model sentence
**Intervention**	≥ 1	Not currently implemented in full-text
**Outcome**	≥ 1	≥ 2 mentions in full text
**Species**	≥ 1	≥ 1 in model sentence
**Sex**	≥ 1	≥ 1 in model sentence

## Data Availability

The underlying data are openly available via the AD-SOLES app or the Open Science Framework project.
