# Supplementary material for: A Systematic Online Living Evidence Summary of experimental Alzheimer’s disease research
Source: J Neurosci Methods. Author manuscript; Available in PMC 2024 Dec 9. (PMC7617163; doi:10.1016/j.jneumeth.2024.110209)
Supplement: Supplementary material [file EMS197993-supplement-Supplementary_material.docx]

Supplementary Figures


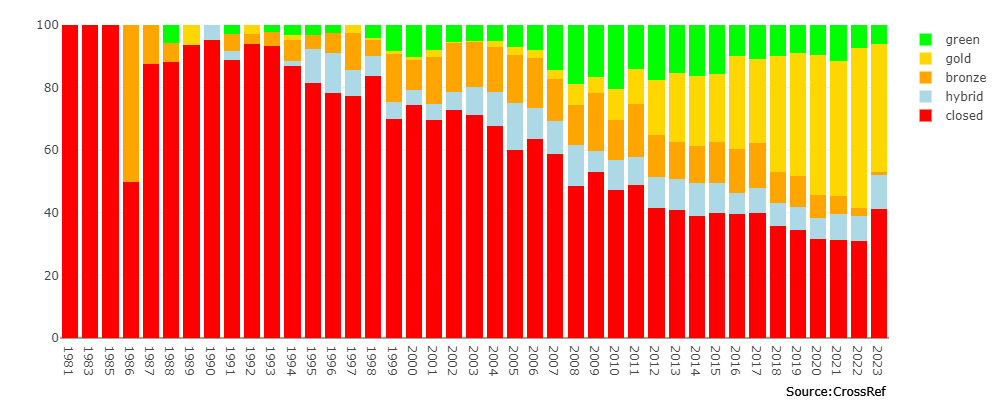


Figure S1: Percentage open access publication types over time in AD-SOLES.
Data source: CrossRef linkage with n= 31,245 articles


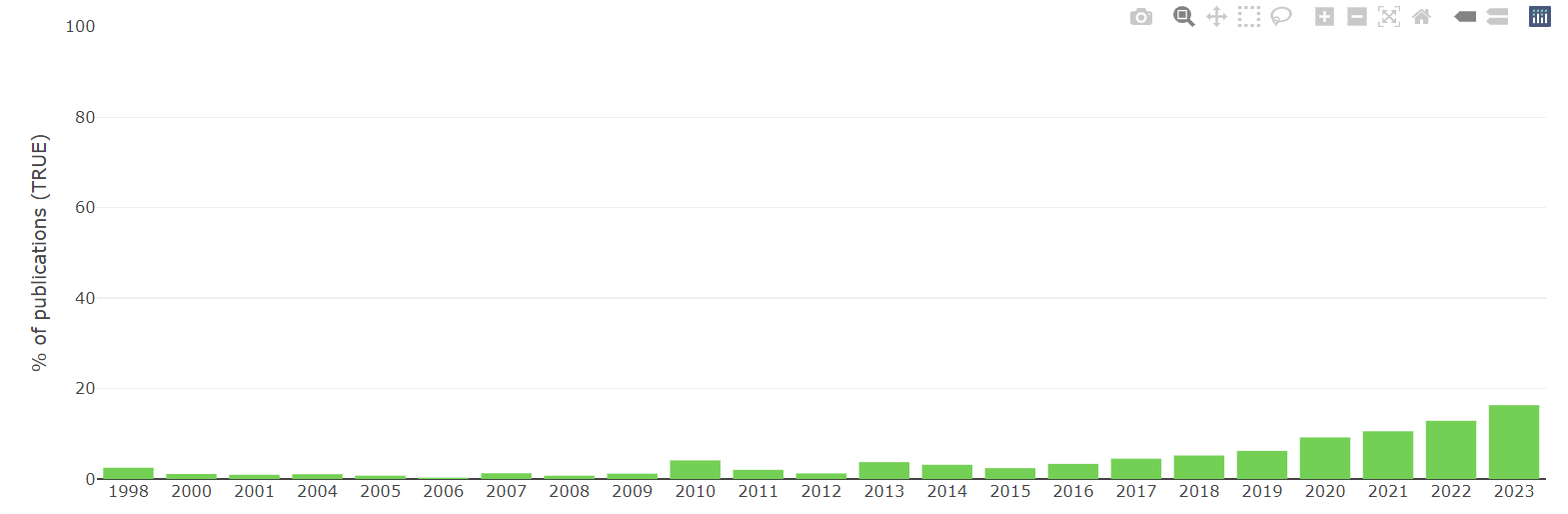


Figure S2: Percentage of publications with open data over time in AD-SOLES.
Tool: ODDPub (48) applied to n=26,920 full text articles.


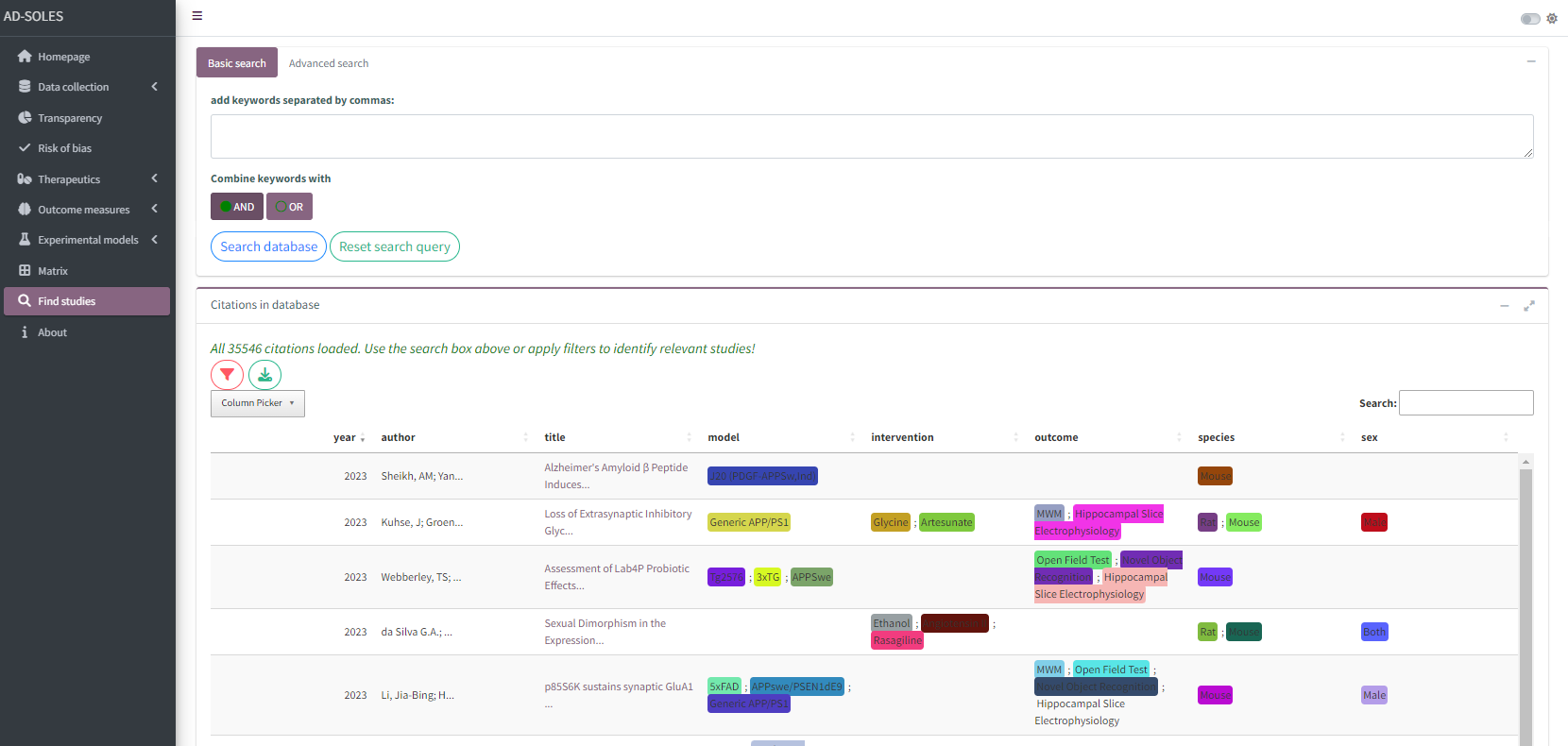


Figure S3: Screenshot of search functionality in AD-SOLES.
